# Genetic and epigenetic analyses of panic disorder in the post-GWAS era

**DOI:** 10.1007/s00702-020-02205-y

**Published:** 2020-05-09

**Authors:** Yoshiro Morimoto, Shinji Ono, Naohiro Kurotaki, Akira Imamura, Hiroki Ozawa

**Affiliations:** 1 Department of Neuropsychiatry, Unit of Translation Medicine, Nagasaki University Graduate School of Biomedical Sciences, Sakamoto 1-7-1, Nagasaki, 852-8501 Japan; 2 Child and Adolescent Psychiatry Community Partnership Unit, Nagasaki University Hospital, Nagasaki, Japan; 3 Aino-Ariake Hospital, Unzen, Nagasaki Japan; 4 Department of Clinical Psychology, Faculty of Medicine, Kagawa University, Kagawa, Japan

**Keywords:** Panic disorder, Genome-wide association studies (GWAS), Missing heritability, Next-generation sequencing (NGS), Rare variant association study (RVAS), Epigenome-wide association study **(**EWAS)

## Abstract

Panic disorder (PD) is a common and debilitating neuropsychiatric disorder characterized by panic attacks coupled with excessive anxiety. Both genetic factors and environmental factors play an important role in PD pathogenesis and response to treatment. However, PD is clinically heterogeneous and genetically complex, and the exact genetic or environmental causes of this disorder remain unclear. Various approaches for detecting disease-causing genes have recently been made available. In particular, genome-wide association studies (GWAS) have attracted attention for the identification of disease-associated loci of multifactorial disorders. This review introduces GWAS of PD, followed by a discussion about the limitations of GWAS and the major challenges facing geneticists in the post-GWAS era. Alternative strategies to address these challenges are then proposed, such as epigenome-wide association studies (EWAS) and rare variant association studies (RVAS) using next-generation sequencing. To date, however, few reports have described these analyses, and the evidence remains insufficient to confidently identify or exclude rare variants or epigenetic changes in PD. Further analyses are therefore required, using sample sizes in the tens of thousands, extensive functional annotations, and highly targeted hypothesis testing.

## Introduction

Panic disorder (PD) is an anxiety disorder characterized by recurrent and unexpected panic attacks, subsequent anticipatory anxiety, and phobic avoidance. Approximately, 10–20% of patients with PD are refractory, with severe and chronic symptoms that seriously affect their quality of life (Markowitz et al. 1989). PD has a high incidence rate, with a lifetime prevalence of 1–3%, and is a disorder frequently observed in clinical practice (Wittchen et al. 2011). Several genetic and epidemiological studies, including family and twin studies, have shown that genetic factors play an important role in the pathogenesis of PD. First-degree relatives of a proband with PD are at a sixfold risk of developing this disease (Goldstein et al. 1997). Additionally, the PD concordance rate is higher in monozygotic twins than in dizygotic twins (Hettema et al. 2001). Conventionally, linkage mapping enables us to identify the causative genetic variants of Mendelian diseases, such as Huntington’s disease, using polymorphic microsatellite markers. However, neuropsychiatric disorders, including PD, are typically not caused by single mutations; therefore, traditional linkage mapping for neuropsychiatric disorders have not achieved the same success as they have for Mendelian diseases (Karayiorgou and Gogos 2006; Venken and Del-Favero 2007). Various candidate gene association studies have been conducted to identify candidate genes for neuropsychiatric disorders, including PD, but few studies have demonstrated reproducible results (Singer 2009; Schumacher et al. 2011).

In recent years, with rapid advances in sequencing technology, large amounts of data can be obtained and various approaches are available to detect disease-causing genes. In particular, genome-wide association studies (GWAS) have attracted attention for their ability to detect disease-associated loci of multifactorial disorders. The GWAS era has been successful in the sense that many loci have been statistically associated with risks for multifactorial diseases, and a large number of these loci have been well replicated (Uniken Venema et al. 2017; Langenberg and Lotta 2018; Dennison et al. 2019).

In the present review, an overview of GWAS of PD is provided, followed by a description of the current and future efforts that are necessary to overcome the remaining issues.

### GWAS of PD

Several GWAS of PD have been published thus far. Otowa et al. (2009) conducted a GWAS in 200 Japanese patients with PD and 200 healthy individuals and identified seven genome-wide significant loci. However, the associations that were found in this 2009 study could not be confirmed in a later reproduction analysis by the same group (Otowa et al. 2010). In the 2010 study, another GWAS was performed, using a larger sample size (718 patients with PD and 1717 controls), and the results of the meta-analysis were reported. Although genome-wide significant single nucleotide polymorphisms (SNPs) were not detected in either the GWAS or the meta-analysis, suggestive associations were observed in several loci, such as the bradykinin receptor B2 (*BDKRB2*) gene (*p* = 1.3 × 10^−5^, odds ratio (OR) = 1.31) (Otowa et al. 2012). Erhardt et al. (2011) conducted a GWAS in 216 patients with PD and 222 controls, and reported that genome-wide significant SNPs were not detected, but that the highest nominal association was found for rs7309727 (*p* = 5.1 × 10^−7^, Cochran–Armitage test for trend: *p* = 7.726 × 10^−7^) in discovery samples. In a subsequent replication analysis with a larger number of samples, they found an association between the two SNP haplotypes (rs7309727 and rs11060369) in the transmembrane protein 132D (*TMEM132D*) gene (*p* = 1.2 × 10^−7^); however, the *p* value did not reach genome-wide significance. Moreover, when comparing a mouse model of extremes in trait anxiety with normal mice, nominally significant differences were observed in *Tmem132d* mRNA expression in the cingulate cortex using a microarray experiment (*p* = 0.004). Additionally, rs13478518, which is located on exon 9 of *Tmem132d*, was reported to be associated with the percentage of time spent in the open arms of the elevated plus-maze test (*p* < 0.01) (Erhardt et al. 2011). The same group conducted a reproduction analysis using an additional PD case–control sample, and found an association between the risk haplotypes and the disorder; however, this association was not observed in an analysis of a Japanese population sample (Erhardt et al. 2012). Additionally, Deckert et al. (2017) conducted a GWAS with a high-dimensional phenotype: PD with and without agoraphobia (AG)-related anxiety phenotype, based on the Agoraphobia Cognition Questionnaire (ACQ). This study was conducted in a sample of 1370 healthy German volunteers, and a genome-wide significant association was found between rs78726293 and rs19126060 in the glycine receptor beta (*GLRB*) gene (rs78726293: *p* = 3.3 × 10^−8^, rs191260602: *p* = 3.9 × 10^−8^). The authors followed up on this finding in a larger dimensional ACQ sample (*n* = 2547) and in independent samples with a dichotomous AG phenotype based on the Symptom Checklist (*n* = 3845) and a case–control sample with the categorical phenotype PD/AG (*n*_combined_ = 1012). Highly significant *p *values were obtained for rs17035816 (*p* = 3.8 × 10^−4^) and rs7688285 (*p* = 7.6 × 10^−5^) in *GLRB*. Furthermore, heterozygous *Glrb* knockout mice showed significantly enhanced agoraphobic behavior, demonstrated by less time spent in the center of the open field test (Deckert et al. 2017). Forstner et al. (2019) conducted a GWAS in 2248 patients and 7992 controls, which is the largest sample size to date, but did not find any loci with genome-wide significant associations. They conducted a follow-up analysis on 255 SNPs (*p* < 1 × 10^−4^) in another independent sample (2408 patients and 22,847 controls) and found that rs144783209 in the SMAD family member 1 (*SMAD1*) gene had the strongest association with PD (*p*_comb_ = 3.10 × 10^−7^) (Forstner et al. 2019).

Table 1 summarizes a list of candidate disease-related loci identified by GWAS.

**Table 1:** Summary of genome-wide association studies (GWAS) of panic disorder (PD)

Study	Sample	Criteria for diagnosis	Genome-wide significant loci in discovery sample	Strongest association in replication sample	Nearby gene	Other phenotypes
Erhardt et al. (2011)	Discovery: 216 PD cases, 222 controls Replication: stage 2: 225 PD cases, 240 controls; stage 3: 468 PD and anxiety cases, 452 controls Country: Germany and European countries	DSM-IV, ICD-10	–	rs7309727 and rs11060369 (*p* = 1.2 × 10^−7^)	*TMEM132D*	MDD (Inoue et al. 2015) Addictive disorders (Hodgson et al. 2016) ADHD (Mick et al. 2011)
Otowa et al. (2012)	Discovery: 718 PD cases, 1717 controls Replication: 329 PD cases, 861 controls Country: Japan	DSM-IV	–	rs10144552 (*p* = 1.3 × 10^−5^)	*BDKRB2*	Substance abuse, BIP, OCD and depression Gratacòs et al. 2009)
Deckert et al. (2017)	Discovery: 1370 controls (dimensional ACQ) Replication: 2547 controls (dimensional ACQ), 3845 controls (dichotomous SCL-90); 506 PD cases, 506 controls Country: Germany and the Netherlands	ACQ, SCL-90	rs78726293 (*p* = 3.3 × 10^−8^) rs191260602 (*p* = 3.9 × 10^− 8^)	rs17035816 (*p* = 3.8 × 10^−4^) rs7688285 (*p* = 7.6 × 10^−5^)	*GLRB*	BIP (Tang et al 2019), ASD (Bonnet-Brilhault et al. 2016)
Forstner et al. (2019)	Discovery: 2248 PD cases, 7992 controls Replication: 2408 PD cases, 228,470 controls Country: Denmark, Estonia, Germany, Sweden, Iceland, and the Netherlands	DSM-III-TR, DSM-IV ICD-10	–	rs144783209 (*p*_comb_ = 3.10 × 10^–7^)	*SMAD1*	–

*PD* panic disorder, *ICD-10* International Statistical Classification of Diseases and Related Health Problems, 10th edition, *DSM-IV* Diagnostic and Statistical Manual of Mental Disorders, 4th edition, *CMC* combined multivariate and collapsing, *SKAT-O* Optimal Sequence Kernel Association Test, *BIP* bipolar disorder, *ADHD* attention-deficit hyperactivity disorder, *SCZ* schizophrenia

## Results and limitations of GWAS

Overall, GWAS have yielded exciting results and revealed unexpected candidate genes that contribute to the risk of PD. Several candidate genes are reported to be associated with other mental disorders, and further functional analyses or clinical applications are expected (Table 1).

To date, however, replicated genome-wide significant findings have not been identified in PD case–control GWAS. Given that genome-wide significant loci have been reported in other anxiety phenotypes (Stein et al. 2017; Meier and Deckert 2019; Purves et al. 2019), the PD GWAS that have so far been reported may be underpowered. An expansion in scale at multiple levels may improve GWAS power. First, because sample size is the primary limitation in risk variant discovery, large sample sizes are expected to result in the identification of additional candidate loci (Visscher et al. 2017). Second, performing GWAS in understudied ethnic groups will be informative, especially for detecting ethnic-specific risk variants (Morris 2011; Li and Keating 2014; Medina-Gomez et al. 2015). Third, the use of innovative GWAS methods and study designs, such as GWAS using Bayesian analysis (Stephens and Balding 2009), GWAS using machine learning approaches (Szymczak et al. 2009), and GWAS using methods to improve power to analyze heterogenous traits, may also lead to the discovery of previously undetected associations (Bhattacharjee et al. 2012).

The value of GWAS lies not only in their utility for identifying disease-associated loci, but also in other applications. For example, polygenic risk scores, which are quantitative measures of risk summed across multiple risk alleles, allow us to separate a population into categories with sufficiently distinct risks, which can then be used for clinical decision-making (Tam et al. 2019). The value of a potential polygenic risk score in mental disorders has been explained in several studies (Gasse et al. 2019; Abdulkadir et al. 2019), and it is expected that this will have future applications in PD. Furthermore, using summary statistics from GWAS, linkage disequilibrium score regression (LDSR) can be used to estimate genetic correlations between separate traits (Bulik-Sullivan et al. 2015). Using LDSR, the genetic correlation between PD and anxiety-related traits has been reported. Otowa et al. (2016) conducted a meta-analysis on nine sample sets (> 18,000 patients) comprising anxiety disorders (ADs), PD, and phobias, and found a significant correlation of genetic risk between ADs and major depressive disorder (MDD), but not between ADs and bipolar disorder or schizophrenia. Forstner et al. (2019) also reported a significant genetic correlation between PD and MDD, depressive symptoms, and neuroticism. These results suggest that ADs, PD, and MDD are likely to have genetic overlap and continuous phenotypes.

The use of GWAS has identified an unprecedented number of genetic variants associated with common disease traits, but aside from a few notable exceptions, these variants account for only a modest proportion of the estimated heritability of most complex traits (Tam et al. 2019). This hidden heritability, known as “missing heritability,” is largely attributed to the limitations of GWAS (Maher 2008). A number of possible explanations for this missing heritability problem include the presence of rare pathogenic variants (Zuk et al. 2014; Bandyopadhyay et al. 2017), epigenetic factors (Trerotola et al. 2015; Bourrat et al. 2017), and the complexity of patient phenotypes (van der Sluis et al. 2010).

In particular, rare variant association studies (RVAS), made possible by the popularization of next-generation sequencing (NGS), are anticipated to cause a paradigm shift to resolve the issue of missing heritability.

### RVAS of PD

NGS has been put into practical use since the early 2000s. With the adoption of sequencing technology that is completely different from conventional Sanger sequencing, the speed of genome sequencing has dramatically increased. With the popularization of NGS, rare genetic variants—variants that are present with a minor allele frequency of less than 1%—with a strong effect size have become identifiable by whole genome sequencing (WGS) and whole exome sequencing (WES). Analyses of neuropsychiatric disorders using NGS have been performed (Sanders 2019; Zhuo et al. 2019), and genes with high ORs have been reported (Fromer et al. 2014; Takata et al. 2014; Singh et al. 2016; McCarthy et al. 2017). NGS is a powerful tool to detect genes that cause not only single-gene disorders, but also multifactorial disorders; however, only a few studies have explored the genes responsible for PD.

Gregersen et al. (2016) conducted WES in 54 patients with PD and 211 controls from a Faroe population. However, no genes had a genome-wide significant association with the disorder. The diacylglycerol kinase eta (*DGKH*) gene demonstrated the strongest association (combined multivariate and collapsing (CMC): *p* = 1.25 × 10^−4^) with PD (Gregersen et al. 2016). Additionally, our group performed WES on one Japanese family, including multiple patients with PD, and identified seven candidate genes. We subsequently conducted an association study on the identified candidate genes using a sample of 952 Japanese and 192 German individuals. Genes demonstrating a significant association with PD were not identified; however, the phospholipase A2 group IVE (*PLA2G4E*) gene had the strongest association (CMC: *p* = 0.0715; Madsen–Browning: *p* = 0.0658; optimal sequence kernel association test: *p* = 0.1624) (Morimoto et al. 2018).

Table 2 summarizes a list of candidate genes identified by RVAS.

**Table 2:** Summary of rare variant association studies (RVAS) of panic disorder (PD)

Study	Sample	Criteria	Gene-based association analysis	Strongest association	Other phenotypes
Gregersen et al. (2016)	54 PD cases, 211 controls Country: Denmark (Faroe Islands)	ICD-10	–	*DGKH* (CMC: *p* = 1.25 × 10^−4^)	BIP (Baum et al. 2008; Zeng et al. 2011; Yosifova et al. 2011), depression (Weber et al. 2011), ADHD (Weber et al. 2011), SCZ (Zeng et al. 2011)
Morimoto et al. (2018)	Discovery: One Japanese family including multiple patients with PD Replication: 477 PD cases, 667 controls Country: Japan and Germany	ICD-10 DSM-IV	–	*PLA2G4E *(CMC: *p* = 0.0715; Madsen–Browning: *p* = 0.0658; SKAT-O: *p* = 0.1624)	–

## Limitations of RVAS

Analyses using NGS have the potential to identify highly pathogenic genetic variants that are difficult to identify using GWAS. In particular, *DGKH* is reported to be associated with other mental disorders, and further clinical applications are expected (Table 2). However, rare variant analysis using NGS has various issues. Genomic variants that substantially contribute to disease development are expected to have very low allele frequencies; therefore, a relatively large sample size is necessary, or the analyses will lack sufficient power to support the study findings (Moutsianas et al. 2015). Additionally, RVAS requires investigators to test a diverse array of genetic hypotheses that differ in the number, frequency, and effect sizes of underlying causal variants. Thus, extensive functional annotation or highly targeted hypothesis testing is also required to be able to confidently identify rare variants in complex diseases (Moutsianas et al. 2015). The sampling of individuals with extreme phenotypes can enrich the presence of causal rare variants, leading to the increased power of RVAS with complex traits compared with random sampling (Barnett et al. 2013).

### Epigenome-wide association studies (EWAS) of PD

Epigenetics is involved in various biological phenomena, including gene expression and cell differentiation (Portela and Esteller 2010). Epigenetic mechanisms, such as DNA methylation, have been suggested to play an important role in disease pathogenesis at the intersection of genetic and environmental factors (Klengel and Binder 2015; Schiele and Domschke 2018), and may be a possible explanation for missing heritability (Trerotola et al. 2015; Bourrat et al. 2017).

Recently, with advances in genome analysis technology, EWAS have become possible (Flanagan 2015). EWAS can be used to analyze genome-wide associations between epigenetic changes and disorders, and several EWAS for multifactorial disorders, including mental disorders, have been reported (Abdulrahim et al. 2019; Starnawska et al. 2019; Gonzalez-Jaramillo et al. 2019).

Iurato et al. (2017) conducted an EWAS in a sample of 89 patients with PD and 76 controls. In the female sample, one locus with genome-wide association was detected in the enhancer region of the hdc homolog, cell cycle regulator (*HECA*) gene (cg07308824: *p* = 1.094 × 10^−7^; *p*-adj = 0.046). Hypermethylation at the same locus was also confirmed in a reproduction analysis (*p* = 0.035; 131 patients with PD and 169 controls). Methylation at this CpG site was associated with *HECA* mRNA expression in another independent female sample (*n* = 71), both at baseline (*p* = 0.046) and after induction by dexamethasone (*p* = 0.029) (Iurato et al. 2017). Furthermore, Shimada-Sugimoto et al. (2017) analyzed DNA methylation levels at CpG sites across the genome in a sample of 48 patients with PD and 48 controls. Forty CpG sites had a significant association with PD, and pathway analysis revealed an association with PD among multiple pathways, including that of positive regulation of lymphocyte activation (Shimada-Sugimoto et al. 2017). Additionally, Ziegler et al. (2019) conducted an EWAS in a sample of 57 patients with PD and 61 controls. Epigenome-wide significant hits were not identified; however, suggestive evidence was observed for decreased methylation at cg19917903 in the cilia- and flagella-associated protein 46 (*CFAP46*) gene. These authors also reported DNA methylation changes as well as clinical effects after 6 weeks of cognitive behavioral therapy in PD, but they did not obtain a hit reaching epigenome-wide significance; however, in patients who responded to cognitive behavioral therapy, there was the most suggestive evidence for methylation at CG06943668 in intron 1 of the interleukin 1 receptor type 1 (*IL1R1*) gene (Ziegler et al. 2019).

Table 3 summarizes a list of candidate disease-related loci identified by EWAS.

**Table 3:** Summary of epigenome-wide association studies (EWAS) of panic disorder (PD)

Study	Sample	Criteria	Tissue	Epigenome-wide significant site	Strongest association	Nearby gene	Other phenotypes
Iurato et al. (2017)	Discovery: 89 PD cases, 76 controls Replication: 131 PD cases, 169 controls Country: Germany	DSM-IV	Blood	cg07308824 hypermethylated (*p* = 1.094 × 10^–7^) Statistical significance: FDR of 5%	cg07308824 Hypermethylated (*p* = 1.651 × 10^–8^)	*HECA*	–
Shimada-Sugimoto et al. (2017)	48 PD cases, 48 controls Country: Japan	DSM-IV	Blood	40 CpG sites Statistical significance: FDR of 5%	cg25270498 Hypomethylated (*p* = 5.67 × 10^–10^)	*METRNL*	–
Ziegler et al. (2019)	57 PD cases, 61 controls Country: Germany	DSM-IV	Blood	– Statistical significance: *p* < 5.77 × 10^–8^	cg19917903 Hypomethylated (*p* = 3.8 × 10^–7^)	*CFAP46*	–

*PD* panic disorder, *DSM-IV* Diagnostic and Statistical Manual of Mental Disorders, 4th edition, *FDR* false discovery rate

## Limitations of EWAS

EWAS have the potential to identify epigenetic changes, which are difficult to detect using conventional technology. In light of the dynamic nature of epigenetic modifications in response to environmental input, recent studies have placed a particular focus on epigenetic changes in relation to the effects of psychotherapy, examining the potential of epigenetic patterns to predict therapeutic correlates of clinical change over the course of psychotherapeutic and preventive interventions (Tomasi et al. 2019; Schiele et al. 2020). As mentioned in the previous section, Ziegler et al. (2019) reported increased methylation at cg06943668 in *IL1R1* in treatment responders after cognitive behavioral therapy; this finding suggests that diagnostic or therapeutic biological markers of PD may be identified by EWAS.

To date, however, a replicated epigenome-wide significant finding has not been identified in PD using EWAS. All EWAS reports have been conducted on small sample sizes and may therefore have low statistical power. Tsai and Bell (2015) simulated the statistical power for case–control and discordant monozygotic twin EWAS study designs, using a range of epigenetic risk effect sizes and conditions. For example, to detect a 10% mean methylation difference between affected and unaffected subjects at a genome-wide significance threshold of *p* = 1 × 10^–6^, 98 monozygotic twin pairs were required to reach 80% EWAS power, and 112 cases and 112 controls pairs were needed for the case–control design (Tsai and Bell 2015).

Additionally, many items require careful consideration in EWAS study design, such as population and tissue selection, population stratification, cell heterogeneity, confounding, temporality, appropriate statistical analysis, and validation of results (Saffari et al. 2018; Michels and Binder 2018). Moreover, it has been recently suggested that differential DNA methylation identified in peripheral blood may not correlate well with methylation changes in brain tissue (Ursini et al. 2011; Provençal et al. 2012; Wang et al. 2012). Further research is therefore needed to investigate whether DNA isolated from peripheral sources can be used as a surrogate biomarker for methylation patterns in the brain.

### Future perspectives

RVAS and EWAS analyses can be adopted to elucidate missing heritability in PD; however, there are only a small number of reports of these analyses, and the evidence remains insufficient. To confidently identify or exclude rare variants or epigenetic changes in complex diseases, further analyses are required using sample sizes in the tens of thousands, extensive functional annotations, and highly targeted hypothesis testing.

Currently used NGS techniques have limitations for identifying structural variants, sequencing repetitive regions, phasing of alleles, and distinguishing highly homologous genomic regions because of its short-read lengths (Mantere et al. 2019). Recently, long-read-NGS (LR-NGS) has emerged, which is expected to improve the characterization of genetic variations and regions that are difficult to assess using prevailing NGS (Lu et al. 2016; Midha et al. 2019). Moreover, several studies have recently demonstrated that LR-NGS technology is ideal for epigenetic characterization (Nakano et al. 2017; Sakamoto et al. 2020). Genetic analyses using the latest technology, including LR-NGS, are expected to lead to the discovery of new PD causative genetic and epigenetic variations.

In the future, methodological progress may contribute to more robust findings that have a strong effect on PD onset and might also contribute to targeted preventive measures and the development of individualized therapeutic approaches. However, because of the higher disease risks that will be implicated in these findings, strict regulations about genetic counseling, confidentiality, and data protection should be applied (Gershon and Alliey-Rodriguez 2013). On a more positive note, the sharing of clinical and genomic data promises to increase research efficiency, expedite translational efforts of research results, and ensure the traceability and transparency of published studies (Shabani and Borry 2015; Takashima et al. 2018). In the post-GWAS era, the ethical framework regarding data sharing should require that researchers and research participants are responsible for protecting privacy, while advancing genomic science and medicine.
